# Circulating exosomes as an enriched source for adult T-cell leukemia biomarkers

**DOI:** 10.1186/1742-4690-12-S1-P33

**Published:** 2015-08-28

**Authors:** Sucharita Dutta, Elizabeth Broughman, Lifang Yang, Scott Peterson, Amol Prakash, O John Semmes

**Affiliations:** 1 The Leroy T. Canoles Jr. Cancer Research Center, Eastern Virginia Medical School, Norfolk, Virginia, USA; 2 Department of Microbiology and Molecular Cell Biology, Eastern Virginia Medical School, Norfolk, Virginia, USA; 3 Thermo Fisher Scientific BRIMS Center, Cambridge, Massachusetts, USA; 4 Optys Tech Corporation, Philadelphia, Pennsylvania, USA

## 

ATL arises from HTLV-1-mediated transformation of infected T-cells following on average 30+ years post initial infection. Current diagnosis relies upon evaluation of numerous clinical factors and is generally effective for overt Acute and Lymphomatous variants of ATL for which survival is less than 1 year post-diagnosis. As the vast majority of infected individuals >95% will remain disease free and as a significant proportion of those that do develop ATL are “curable/treatable” there is a clear need for early definitive diagnosis of HTLV-1-infected individuals. We hypothesized that immune evasion by HTLV-1 and crosstalk between complement, fibrinolysis and coagulation (critical to hematopoietic stem cell renewal) provide pathway-specific biomarker events that are concentrated in circulating exosomes. We subjected exosomes isolated from serum of HTLV-1-infected patients to quantitative ultra-high resolution mass spectrometry that identified 784 proteins of which 208 displayed significant expression differences. We further analyzed our data with customized algorithms designed to facilitate a targeted analysis of the potential biomarker candidates inclusive of complex post-translational modifications. This approach allowed for high confidence quantitation of a panel of proteins that effectively discriminate between AS/HAM/ATL. We observed stratification of the disease states across the complement superfamily, over expression of the coagulation proteolytic cascade in HAM/TSP and ATL, and over expression of alpha-1-antitrypsin in ATL. When compared to a parallel analysis of total serum, exosomes reproducibility reflected expected tumor cell phenotype. For example, plasminogen activator inhibitor was over expressed; where as fibrinogen and metalloproteinases were downregulated in ATL exosomes. This is consistent with the function of PAI-1 to inhibit fibrinogen and MMPs. Interestingly, PAI-1 is not normally over expressed in hematopoietic disorders except Acute Lymphocytic Leukemia. We also identified APOBEC3H, which has been reported to bind specifically to HTLV-1 gag, as over expressed in ATL. We also examined the exosomes for evidence of HTLV-1 proteins. We were able to identify Tax and to a lesser extent Hbz each showing a trend of no-expression in AS, detectable expression in HAM/TSP and higher expression in ATL.

